# Paratesticular Cellular Angiofibroma (CAF): A Rare Case Report

**DOI:** 10.7759/cureus.43124

**Published:** 2023-08-08

**Authors:** Sofia Gaspar Reis, Duarte Gil Alves, Sara Anacleto, Nuno Mendonça, Hélder Além

**Affiliations:** 1 General Surgery, Centro Hospitalar Barreiro Montijo, Barreiro, PRT; 2 General Surgery, Hospital Dr. Nélio Mendonça, Funchal, PRT; 3 Urology, Hospital de Braga, Braga, PRT

**Keywords:** angiomyofibroblastoma-like tumor, benign urological tumor, mesenchymal tumor, paratesticular tumor, cellular angiofibroma

## Abstract

Cellular angiofibromas (CAFs) are infrequent and benign soft-tissue tumors that primarily affect the genitourinary region in both genders. The authors report the case of a 71-year-old male patient who exhibited progressively increasing swelling in both testicles, with greater prominence noted on the left side. Initial findings from physical examination and scrotal ultrasound indicated the possibility of bilateral hydrocele, so the patient was recommended surgical intervention of the left more prominent side. Intraoperatively, a left paratesticular mass was identified and subsequently excised. Histopathological analysis confirmed the diagnosis of cellular angiofibroma. Surgeons should be cognizant of this tumor type to optimize treatment strategies, as local excision demonstrates a potential to preserve the testicle and yield favorable outcomes. Although occurrences of local recurrence are extremely rare, long-term follow-up is imperative.

## Introduction

Cellular angiofibromas (CAFs) are uncommon benign soft-tissue tumors that predominantly manifest in the genitourinary region. These tumors affect both genders, typically appearing during the fifth decade of life [1]. In women, CAFs primarily occur in the vulvo-vaginal region, while in men, they are more commonly found in the inguino-scrotal area [2-3]. However, it is worth noting that extragenital localizations of CAFs have also been reported [1, 4-5]. The precise pathogenesis of CAFs remains to be fully elucidated.

Histopathologically, CAFs are primarily characterized by bland spindle cells containing short bundles of wispy collagen. Additionally, small- to medium-sized vessels with mural hyalinization are commonly observed. Neoplastic cells in CAFs demonstrate positive expression of CD34 and epithelial membrane antigen (EMA). Chromosomal analysis has indicated the potential presence of RB1 deletion on chromosome 13q14 [6-8].

Clinically, CAFs can be challenging to suspect due to their association with conditions such as hydrocele testis and/or inguinal hernias. The definitive diagnosis of cellular angiofibroma is established through histological examination, and the tumor's location serves as an essential clue in the diagnostic process [6].

Complete excision represents the sole curative treatment [8-9]. To date, the literature suggests that recurrence of CAF has only been reported in a single documented case [10], and there have been no reported instances of metastasis. Since the initial descriptions of CAFs [2-3], only a limited number of studies have been published in the literature. These predominantly consist of single case reports or reviews encompassing cases from both genders. In this article, the authors present a case study of a paratesticular cellular angiofibroma that was effectively treated through excision.

## Case presentation

A 71-year-old Caucasian male patient was referred to the surgical department with complaints of swelling in the left testicle that started four months after a left inguinal hernioplasty. The patient had previously undergone hernioplasty as an outpatient, using the plug and mesh technique. Following an ultrasound study, the urology consultation diagnosed bilateral hydrocele, with the left side exhibiting a more pronounced swelling. As the patient had already been operated on by General Surgery, it was agreed with the Urology department that the general surgeons would do the hydrocele corrective surgery.

The patient's medical history included a previous diagnosis of colorectal adenocarcinoma (T3N1M0), for which he had undergone rectum anterior resection and adjuvant chemotherapy two years prior and was considered cured. Additionally, he had a medical history of atrial fibrillation, arterial hypertension, hyperuricemia, dyslipidemia, and class I obesity (BMI 31 kg/m2), medicated with Rivaroxaban, Ramipril, Allopurinol, and Simvastatin. There were no relevant family history details pertaining to this case.

Investigations

Physical examination revealed asymmetrical enlargement of both testicles, with the left scrotum demonstrating a more significant increase in volume. Transillumination and fluctuation tests were positive bilaterally, while impulse on coughing was negative. Palpation of the testicles did not elicit any pain. No other findings were reported on urogenital and rectal examination. Ultrasound examination confirmed the presence of simple fluid collection on both sides. No masses or alterations were detected. Doppler sonography was not performed.

Treatment

**Figure 1:**
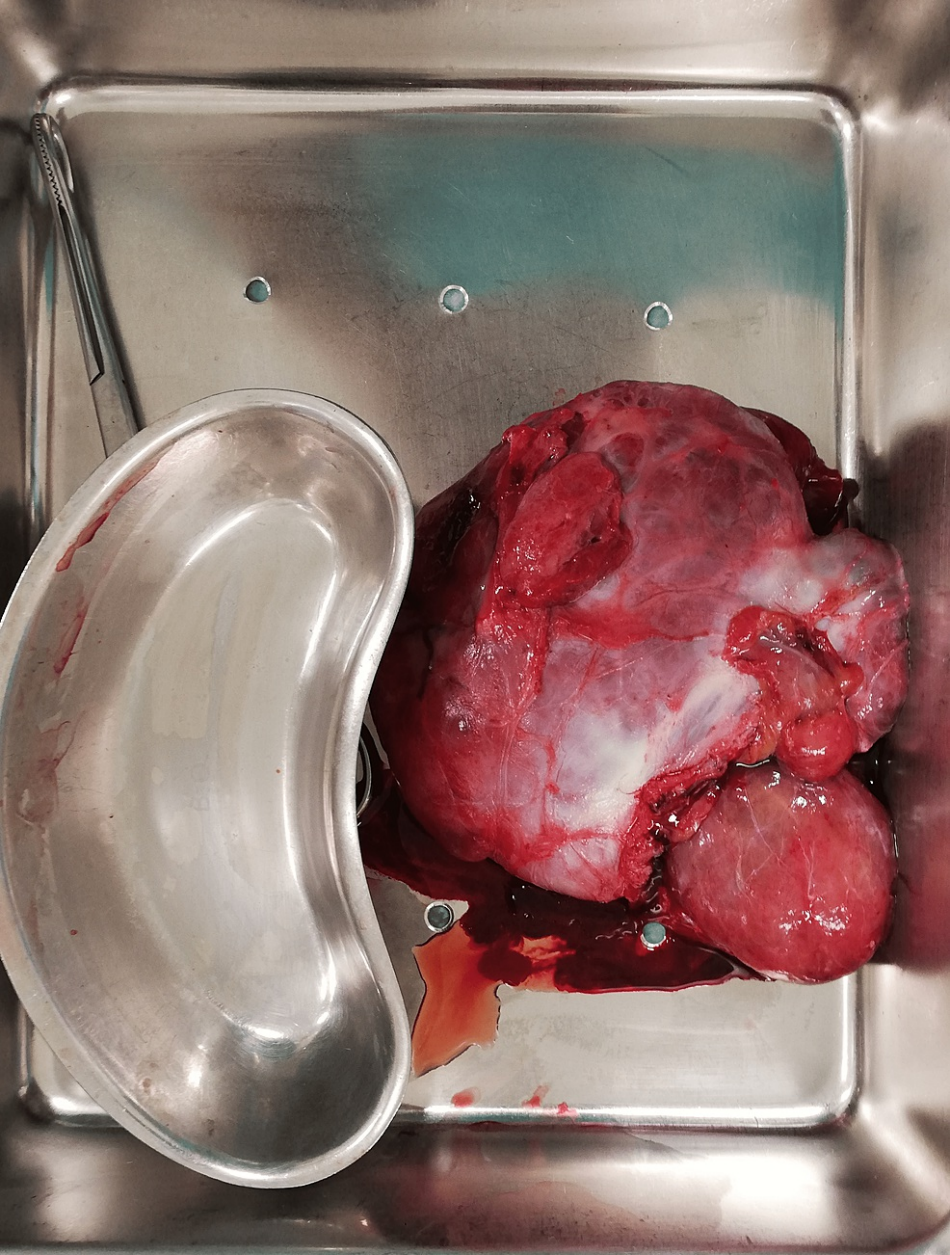
Surgical specimen.

**Figure 2:**
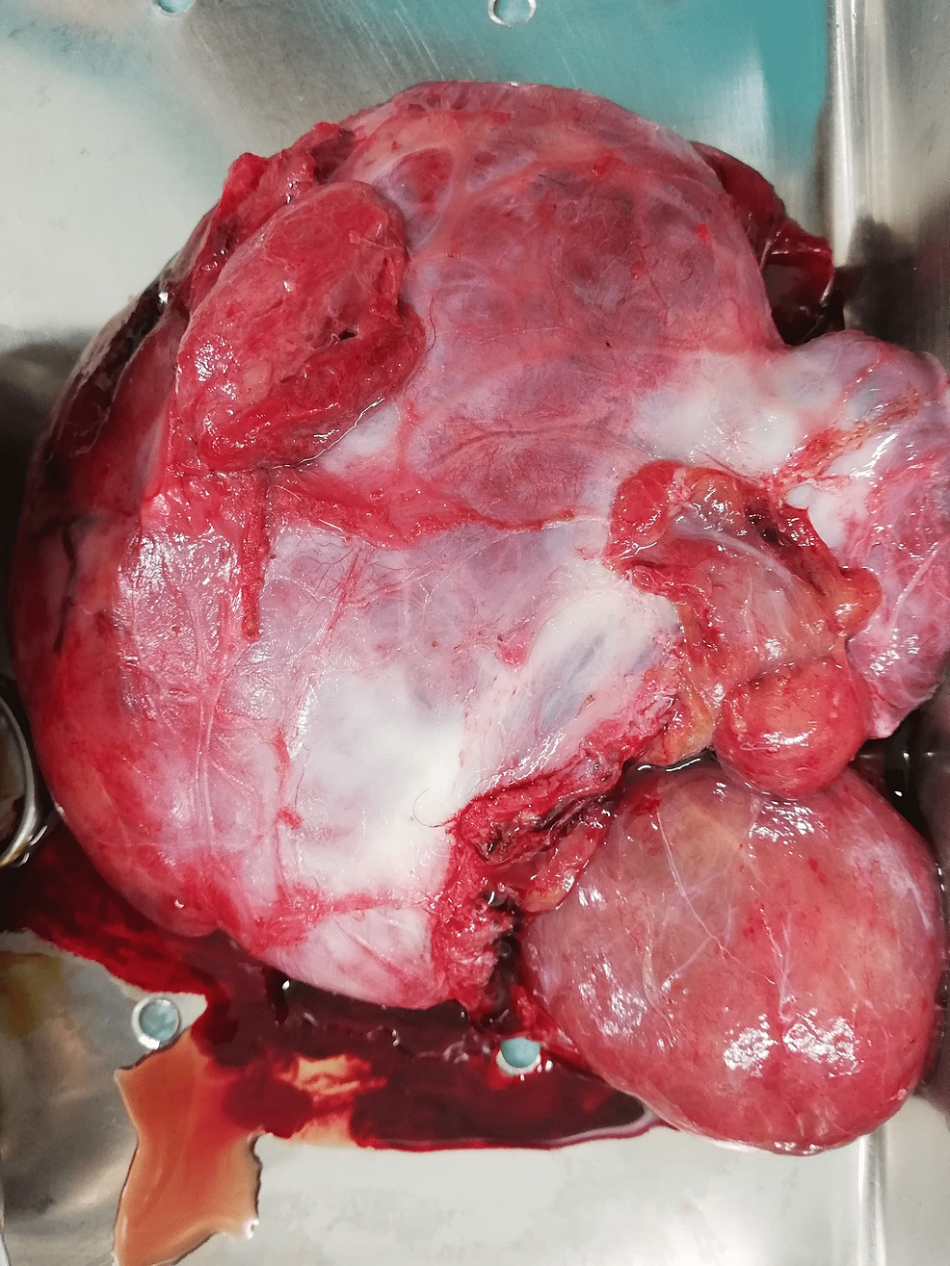
Surgical specimen in detail.

The patient provided consent for drainage of the left hydrocele and eversion of the tunica vaginalis. During the procedure, an oval-shaped soft paratesticular mass measuring approximately 10 cm × 10 cm × 4 cm with rich vasculature was found. The mass appeared to be attached to, but not invading, the scrotal part of the spermatic cord. The tumor was completely resected (Figures 1-2), and hydrocele drainage was performed. The testis, epididymis, and spermatic cord were inspected and found to be intact without any evidence of injury. They were carefully repositioned in the scrotum in their normal anatomical position, ensuring that the spermatic cord was not twisted. To prevent hydrocele recurrence, eversion of the tunica vaginalis was performed using Jaboulay's technique.

Outcome and follow-up

Histopathology reports described a nodular fragment measuring 300 g and 11 cm × 12 cm × 4 cm. The outer surface was partially covered with a fibrous membrane, while the remaining surface had a fleshy, grayish-brown appearance. Upon sectioning, the surface displayed a vaguely fatty appearance with slightly hemorrhagic areas and regions of increased consistency.

Microscopically, the lesion exhibited expansive growth without an obvious peripheral capsule. It consisted of numerous small to intermediate-sized vascular structures within a typically lax stroma. The cellular component demonstrated oval nucleus elements without significant atypia, with discrete mitotic activity (Ki67 less than 5%). The vascular structures often displayed hyalinized walls and occasional intravascular thrombosis. In other sections, the neoplasm resembled granulation tissue with marked interstitial edema surrounding the vascular structures. Proliferating cells showed immunoreactivity for Bcl2 and EMA.

There were no postoperative complications, and the patient was discharged on the first postoperative day. Regular follow-up examinations were conducted, and there were no reports of recurrence during the two-year period. However, after the two-year follow-up, the patient, unfortunately, succumbed to septic shock of respiratory origin.

## Discussion

Cellular angiofibroma is a rare mesenchymal tumor characterized by its generally well-circumscribed shape [8].

Patients with CAF typically present with a slow-growing painless mass, with a median duration of 5 months [1]. However, some individuals may experience mild-to-moderate pain or symptoms related to the surrounding tissues [1, 11-12]. The median size of the tumor is 2.8 cm for women and 7 cm for men, and the cut surface typically appears gray-pink-brown [1, 12].

Clinically, these tumors can be misdiagnosed as an inguinal hernia, despite not exhibiting pulsation during the Valsalva maneuver. In this specific case, despite not having been operated on for the first time in the authors’ department, it is possible that the patient never had an ipsilateral inguinal hernia, and if so, the testicular mass may have gone unnoticed due to its small dimensions. Another specific aspect of this case was the presence of bilateral hydrocele, which served to camouflage the mass, probably significantly smaller at the time of the scrotal ultrasound. Therefore, it is crucial to conduct a careful and thorough objective examination along with an ultrasound study. In cases of doubt, further investigation should be pursued, even for small masses.

Diagnostic imaging modalities for CAFs include ultrasonography with color Doppler, CT scan, and MRI. The appearance of cellular angiofibroma on imaging studies can vary, with some tumors demonstrating hyperintense signals on T2-weighted MRI images or density changes on CT scans due to their vascularity [13-14]. However, radiological findings are not highly specific and do not significantly contribute to the differential diagnosis among other mesenchymal tumors.

Histologically, CAFs exhibit distinctive features. These tumors are typically well-circumscribed, cellular, and composed of spindle-shaped cells with short bundles of collagen, fusiform nuclei, and pale eosinophilic cytoplasm. Notably, numerous thick-walled and hyalinized vascular structures are observed. Cellular atypia or necrosis is not commonly detected [15].

Immunohistochemically, CAFs typically show positive staining for vimentin, CD34, and estrogen receptor (ER), while they are negative for desmin, smooth muscle actin (SMA), S100 protein, and epithelial markers. The presence of CD34 and ER immunopositivity further supports the vascular and hormonal characteristics of CAFs. These distinctive histological and immunohistochemical features aid in the accurate diagnosis and differentiation of CAFs from other spindle cell tumors and help guide appropriate clinical management [1].

The diagnosis of CAF is typically based on several key factors, including its specific anatomical location in the vulva-vaginal and inguinal-scrotal regions, the histopathology of spindle-shaped cells, the expression of specific marker proteins by tumor cells, and the absence of one of the two RP1 genes. Distinguishing CAF from two other spindle-shaped cell tumors, namely myofibroblastoma and spindle cell lipoma, can be challenging, as these tumors also commonly exhibit tumor cells with deletions in one of their two RP1 genes. However, certain distinguishing features such as the presence of hyalinized blood vessels and the expression of CD34 and desmin proteins in cellular angiofibroma can be useful in setting it apart from myofibroblastoma [16].

Once the diagnosis of CAF is established, the treatment of choice is surgical excision with tumor-free margins. In this case, at the time of surgery, a definitive diagnosis was not available. However, since the mass was easily isolated from the testicular and peri-testicular structures, a decision was made to spare these organs. Two urologists were consulted during the procedure, and they agreed with the surgical approach [11].

## Conclusions

In conclusion, this case report presented a rare occurrence of CAF. The diagnosis of this type of tumor is challenging due to its nonspecific clinical and radiological features, often leading to misdiagnosis or unnecessary invasive procedures. The case highlights the importance of accurate histopathological evaluation and immunohistochemical analysis to confirm the diagnosis. While surgical excision remains the mainstay of treatment for CAFs, long-term follow-up is necessary to monitor for any recurrence or associated complications. Future research and case studies are needed to further explore cellular angiofibroma's pathogenesis, molecular characteristics, and potential therapeutic targets.
